# A Machine Learning Predictive Model of Bloodstream Infection in Hospitalized Patients

**DOI:** 10.3390/diagnostics14040445

**Published:** 2024-02-17

**Authors:** Rita Murri, Giulia De Angelis, Laura Antenucci, Barbara Fiori, Riccardo Rinaldi, Massimo Fantoni, Andrea Damiani, Stefano Patarnello, Maurizio Sanguinetti, Vincenzo Valentini, Brunella Posteraro, Carlotta Masciocchi

**Affiliations:** 1 Dipartimento di Scienze di Laboratorio e Infettivologiche, Fondazione Policlinico Universitario A. Gemelli IRCCS, 00168 Rome, Italy; 2 Dipartimento di Sicurezza e Bioetica, Università Cattolica del Sacro Cuore, 00168 Rome, Italy; 3 Dipartimento di Scienze Biotecnologiche di Base, Cliniche Intensivologiche e Perioperatorie, Università Cattolica del Sacro Cuore, 00168 Rome, Italy; 4 Real World Data Facility, Gemelli Generator, Fondazione Policlinico Universitario A. Gemelli IRCCS, 00168 Rome, Italy; 5 Dipartimento di Diagnostica per Immagini, Radioterapia, Oncologia ed Ematologia, Fondazione Policlinico Universitario A. Gemelli IRCCS, 00168 Rome, Italy; vincenzo.valentini@unicatt.it; 6 Dipartimento di Scienze Radiologiche ed Ematologiche, Università Cattolica del Sacro Cuore, 00168 Rome, Italy; 7 Dipartimento di Scienze Mediche e Chirurgiche Addominali ed Endocrino Metaboliche, Fondazione Policlinico Universitario A. Gemelli IRCCS, 00168 Rome, Italy

**Keywords:** bloodstream infections, machine learning, prediction

## Abstract

The aim of the study was to build a machine learning-based predictive model to discriminate between hospitalized patients at low risk and high risk of bloodstream infection (BSI). A Data Mart including all patients hospitalized between January 2016 and December 2019 with suspected BSI was built. Multivariate logistic regression was applied to develop a clinically interpretable machine learning predictive model. The model was trained on 2016–2018 data and tested on 2019 data. A feature selection based on a univariate logistic regression first selected candidate predictors of BSI. A multivariate logistic regression with stepwise feature selection in five-fold cross-validation was applied to express the risk of BSI. A total of 5660 hospitalizations (4026 and 1634 in the training and the validation subsets, respectively) were included. Eleven predictors of BSI were identified. The performance of the model in terms of AUROC was 0.74. Based on the interquartile predicted risk score, 508 (31.1%) patients were defined as being at low risk, 776 (47.5%) at medium risk, and 350 (21.4%) at high risk of BSI. Of them, 14.2% (72/508), 30.8% (239/776), and 64% (224/350) had a BSI, respectively. The performance of the predictive model of BSI is promising. Computational infrastructure and machine learning models can help clinicians identify people at low risk for BSI, ultimately supporting an antibiotic stewardship approach.

## 1. Introduction

Hospital-acquired (HA) bloodstream infection (BSI) is a frequent and challenging clinical condition worldwide with a documented considerable impact on hospitalization length and healthcare costs. In 2019, a systematic review of the literature estimated 4.95 million deaths associated with bacterial antimicrobial resistance (AMR) and 1.27 million deaths attributable to bacterial AMR, BSI being the second most frequent infectious syndrome [1].

Without treatment, evolution from BSI to sepsis, a complex life-threatening syndrome caused by a dysregulated host response to infections, is highly probable [2,3]. As pooled mortality of sepsis can reach 40% in critically ill patients [4,5], the early recognition of sepsis and initiation of antibiotic and support therapies are recommended to increase life expectancy [6]. On the other hand, clinical recognition of BSI or sepsis can be challenging. Therefore, clinicians, guided by the fear of an unfavorable evolution of the patient, are prone to prescribe empirical antibiotic overtreatments with possible consequences such as adverse drug effects, the emergence of multidrug-resistant infections or *Clostridioides difficile* infection, and an increase in healthcare costs [7], rather than opting for a watchful waiting approach [8] to save useless antibiotics when BSI is not confirmed. Given that antibiotic therapy is lifesaving and, at the same time, the major driver of antibiotic-resistant microorganism selection, it is of great importance to distinguish those clinical situations for which the initiation of antibiotic therapy should be started as soon as possible from those for which it could be delayed or even avoided.

Machine learning (ML)-based approaches are considered more accurate than traditional clinical scores [9,10], thanks to the larger amount of data split into two different datasets (training and validation) and the flexible nature of the algorithm. While numerous ML-based predictive models for early sepsis identification have been developed, showcasing impressive accuracy, those specifically designed for patients with BSI are relatively scarce. Some studies have focused on the prediction of mortality of patients with BSI. The Bloomy score [11] demonstrated a good performance in predicting the 14-day and 6-month mortality of an ML model on patients with BSI caused by multidrug-resistant organisms. Other studies showed similar good performance of ML-based models for the prediction of mortality in people with BSI [10,12,13,14]. Few studies investigated the accuracy of models to predict the probability of having a BSI in people undergoing blood cultures (BCs). These models demonstrated acceptable performances in patients admitted to the triage of an Emergency Department [15,16], in patients admitted to the Intensive Care Unit [17], in febrile children [18], and in hemodialysis patients [19]. In a study of an ML-based model, the authors predicted BSI and estimated the performances in subgroups stratified by causative pathogen, where *Acinetobacter baumannii, Escherichia coli*, and *Klebsiella pneumoniae* showed high accuracy for bacteremia prediction [20].

Therefore, the objective of the study was to build a predictive model for HA-BSI in patients admitted to ordinary wards. To achieve this aim, we conducted the following: (i) several parameters were automatically extracted from electronic health records (EHR) to build a multidimensional BSI Data Mart; (ii) a machine learning-based pipeline was implemented, and several predictors of HA-BSI were identified; (iii) we assigned a probabilistic risk level for HA-BSI (categorized as low or high risk) for each hospitalization. This predictive model serves to support an antibiotic stewardship approach in managing and optimizing the use of antibiotics.

## 2. Materials and Methods

### 2.1. BSI Data Mart Building Procedures

The implementation of the ML-based pipeline was performed by the Generator Center at the Fondazione Policlinico Universitario A. Gemelli IRCCS (FPG), Rome, Italy. The Generator Real World Data (RWD) lab is responsible for extracting, standardizing, and integrating the huge amount of both structured and unstructured healthcare data, which are heterogeneously stored in the hospital’s Data Warehouse (DWH) and in the archives of individual departments. Both ontology-based systems and information technology (IT) procedures were implemented to build an integrated pseudo-anonymized database, ensuring data ownership and patient privacy. Then, predictive models were developed and implemented with the aim of supporting clinical diagnosis [21]. The first step of the pipeline construction was the development of an integrated and multidimensional database (Data Mart). The Data Mart was based on an ontology defined by an interdisciplinary group of clinicians, who identified an extensive list of variables usually considered in clinical practice and in the treatment of possible BSI. Starting from these selected variables, the corresponding data sources in the EHR archives were identified. Specific extraction, transformation, and loading (ETL) procedures were developed for structured and free-text reports to integrate all the relevant data sources and save the variables under study in a single multidimensional database. In the case of structured variables, such as laboratory analyses and unique identification codes, material and units of measurement were defined and extracted for each variable. For the variables generally reported in free-text clinical reports, such as clinical/nursery diaries, a dedicated extraction process was developed. Natural Language Processing (NLP) was implemented based on text mining procedures, including sentence/word tokenization, a rule-based approach supported by annotations defined by clinical experts, and the use of semantic/syntactic corrections. The Data Mart was developed using the SAS Institute software analysis tool, the SAS^®^ Vyia^®^ environment V.03.05. The Open-Source R^®^ environment version 4.0.4 was used for rapid prototyping and modeling.

### 2.2. Ontology and Study Design

The Data Mart consists of all patients for whom BCs were performed during the hospitalization. All BCs performed from peripheral access (PA) and/or central venous catheter (CVC) during the same calendar day were considered representative of a single event. Only the first event per single patient, i.e., collected within the first 24 h of the onset of signs and symptoms, was included, whereas those following the initial event were a priori excluded. Second and further episodes of BSI during the same hospitalization were excluded because the risk of further BSI could have been influenced by the first episode. The presence of BSI was defined as the growth of a clinically relevant microorganism in at least one BC or as the growth of a potential contaminant (i.e., coagulase-negative staphylococci, *Bacillus* spp., viridans group streptococci, *Corynebacterium* spp., *Propionibacterium* spp., and *Micrococcus* spp.) in multiple BCs. For each hospitalization, demographics, comorbidities, vital signs, devices, and laboratory data were queried from the Data Mart. The information gathered on clinical signs, devices, and laboratory values was closest to the date of the BC request. Specifically, only values recorded within two days before or after the BC date were considered. Preference was given to data on the same day as the request or on previous days.

### 2.3. Cohort Selection

**Figure 1:**
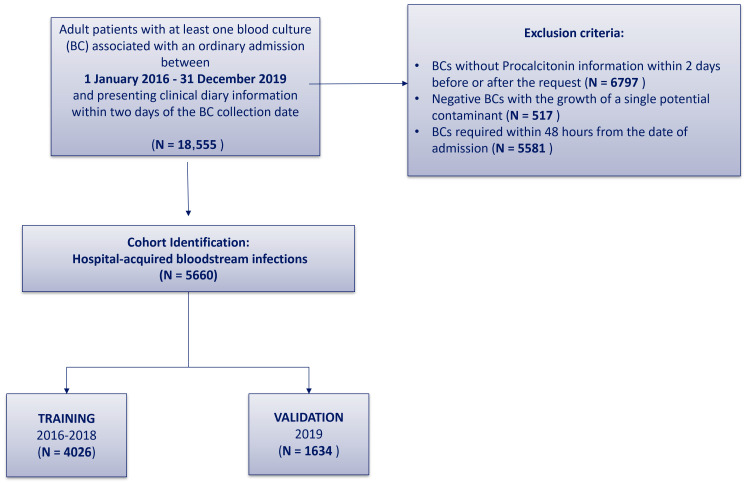
Cohort selection.

To identify clinical predictors of BSI, hospitalizations were selected from the entire Data Mart with the aim of analyzing as homogeneous a group as possible. Cohort selection criteria were defined by clinicians and summarized in Figure 1. The cohort included all patients (age ≥ 18 years) hospitalized at FPG for whom BCs were performed within the period from 1 January 2016 to 31 December 2019 and presenting clinical diary information within two days from the date of BC collection. Each patient was included at the time of the first negative or positive BC during the study period. All BCs without procalcitonin information within two days of the BC collection date were excluded from the analysis. Moreover, single BCs with contaminants were excluded. To have a cohort as homogeneous as possible, only hospital-acquired BSI (HA-BSI) were considered for the study. Therefore, all BCs performed within the first 48 h of hospital admission were excluded.

### 2.4. Statistical Analysis

**Figure 2:**
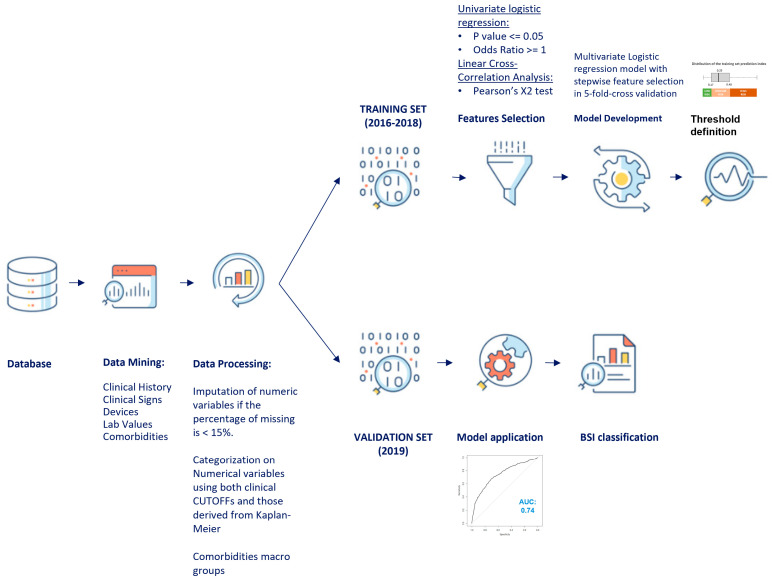
Pipeline for the predictive model of hospital-acquired bloodstream infection (BSI).

The pipeline for the BSI prediction model is illustrated in Figure 2. All variables included in the study were first analyzed by descriptive statistic techniques. Qualitative variables were described as absolute and relative frequencies. Quantitative variables were described as medians and interquartile ranges (IQR). Numerical variables with a percentage of missing data < 15% were imputed with the median imputation technique. To understand the effect of imputation, the distribution of the percentages of missing values and the percentage of change in the correlation coefficient with respect to the patients with or without BSI of the imputed and not imputed variables was analyzed. Comorbidities were aggregated by means of an index considering 5 macro-groups (Index CM). The macro-groups were immunodepression (composed of one or more among cirrhosis, connective tissue disease, HIV, autoimmune disease, transplantation), neurological pathology (composed of one or more among neurological disease, dementia, hemiplegia, stroke, cerebrovascular disease), neoplasia (composed of one or more among cancer, hematological neoplasia, leukemia, lymphoma, or treatment with chemotherapy or radiotherapy), renal insufficiency (kidney failure and/or dialysis), and diabetes. The belonging to each group had the same weight; the index was given by the sum of the membership in a macro-group and ranged from 1 to 5. A univariate statistical analysis was performed on the entire dataset using the chi-square test for categorical variables and the Mann–Whitney test for numerical variables. The numerical variables were made categorical using both cut-offs derived from clinical practice and those derived from the distribution of each variable based on the BSI outcome. In particular, the Kaplan–Meier estimator was used to identify the value that maximizes the statistical difference between positive and negative BSI. All cut-offs were then clinically validated. Several steps were followed to build the model. Initially, the entire dataset was divided into two groups for training and model validation, respectively. In the training group, a univariate analysis was performed to select features by evaluating the relationship of each variable with the outcome (univariate logistics), and then the correlation between variables was analyzed to remove redundancy. Multivariate logistic regression was used to construct a clinically interpretable multivariate predictive model. Logistic regression is considered a standard machine learning model in the clinical setting, as it has shown a good trade-off between predictive performance and clinical interpretability in various contexts [22,23]. The different steps have been summarized in Figure 2 and detailed below.

The initial dataset was divided into two groups for model training and validation based on the BC request date. The model was trained in the first three years of data (1 January 2016–31 December 2018) and tested in the next year (1 January 2019–31 December 2019). A univariate logistic regression was first used on the training set to select candidate predictors of BSI. The *p*-value and Odds Ratio (OR) were estimated, and only the features with *p-*value ≤ 0.05 and Odds Ratio > 1 were included. A linear cross-correlation analysis (Pearson’s correlation method) of the significant variables was performed to exclude linearly related variables. Among the pairs of linearly correlated variables (Pearson’s correlation coefficient > 0.8), we removed variables with lower correlation with the outcome of interest. A multivariate logistic regression with stepwise feature selection in five-fold cross-validation was applied to the training set to express the risk of BSI. The out-of-sample performance of the model was initially estimated on the training set as internal validation in terms of area under the receiving operator curve (AUROC) and as statistics of the classification matrix by averaging the results of the fivefold cross-validation. The same metrics were calculated on the test set to evaluate the performance of the model on a different set of patients from the training set. A calibration plot was also elaborated to estimate the accuracy of the model using Hosmer and Lemeshow goodness-of-fit (GOF) test, and *p*-values < 0.05 indicated lack of fit of the model. Finally, an analysis of lift and gain graphs was reported to identify segments of outcome probability where the model proves to be useful compared to not having the model. Based on the median distribution of the probabilistic predictive values of the training set, the statistics of the classification matrix were calculated, including accuracy to quantify the percentage of instances classified correctly among all instances, sensitivity and specificity to measure the proportion of actual positive or negative instances correctly identified by the classifier, respectively, positive predictive value (PPV) and negative predictive value (NPV). The threshold was estimated on the training set and applied to the validation set. Three risk classes (low, medium, and high risk) were identified based on quartiles of the probabilistic distribution of the prediction. Specifically, validation prediction values below the first quartile were considered as low risk, values between the first and third quartile as medium risk, and values above the third quartile as high risk of BSI. Data analysis was performed with R version 3.6. Data was stored in SAS Viya V.03.05 and accessed through R with SWAT library version 1.5.0. The study was done according to TRIPOD guidelines and should be considered a TRIPOD 2b since it includes a training and testing phase from a single institution [24,25].

## 3. Results

The eligible training cohort included a total of 5660 hospitalizations with at least one BC request during the timeframe from 1 January 2016 to 31 December 2019, more than 48 h from the hospital admission. In the entire cohort, the number of hospitalizations with HA-BSI was 1904 (33.6%). The numerical variables that were imputed were as follows: white blood cells, platelets, creatinine, blood urea, neutrophils, C-reactive protein (missing value < 8%), and total bilirubin (missing value < 15%). The percentage of missing values in the positive and negative population is comparable (difference <2.25% for all variables), and the percentage difference in the correlation coefficient with respect to patients with and without BSI of the imputed and non-imputed variables is < 10% for all variables analyzed. Clinical and demographic information of the study population is shown in Table 1. All numerical variables were categorized according to the cut-offs described in Table S1.

The dataset was divided into two groups for training and validation: 4026 (71%) hospitalizations with BC requests between 1 January 2016 and 31 December 2018 were used to train the model, while 1634 (29%) hospitalizations with BC requests between 1 January 2019 and 31 December 2019 were used to test the model. The occurrence of HA-BSI was 34.0% for the training set and 32.7% for the test set. Characteristics of the population belonging to the two groups are shown in Table 1. The subset of univariate significant variables and the final predictors of the multivariate analysis are shown in Table 2. Age > 80 years, fever, hypotension, altered mental status, central venous catheter, blood urea nitrogen > 13 mg/dl, procalcitonin > 1 ng/mL, total bilirubin > 2 mg/dl, time from admission to BSI > 12 days, 2 or more index comorbidities and platelets < 50,000/mm^3^ were associated with an increased risk of having a BSI.

Five-fold cross-validation resulted in an AUROC of 0.74 on the training set. The respective statistics of the classification matrix are shown below: accuracy 0.66, sensitivity 0.74, specificity 0.62, NPV 0.82, and PPV 0.50. The model was then tested on the validation set. The performance in terms of AUROC was 0.74, and the confusion matrix was as follows: accuracy 0.69, sensitivity 0.69, specificity 0.69, NPV 0.82, and PPV 0.52. No statistically significant difference (*p* > 0.05) was observed between actual and predicted BSI and the corresponding calibration plot, as shown in Figure S1. A lift and gain analysis of the validation set is shown in Figure S2. The lift plot on the testing data showed that for the first decile of predictions, the model performs more than two times better than random guessing based on prevalence only.

**Figure 3:**
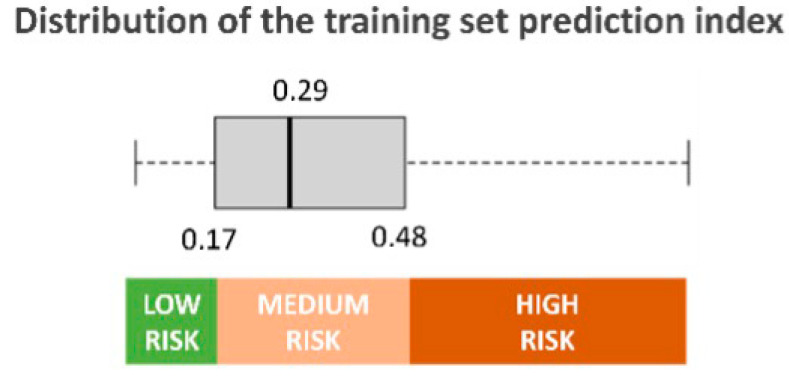
Distribution of the training set prediction index and definition of risk classes.

Three risk groups were selected based on the interquartile predicted risk score on the training set to better categorize the patient risk and minimize antibiotic treatment of those without a true BSI. Of the entire validation set, 508 (31.1%) patients were classified at low risk, 776 (47.5%) at medium risk, and 350 (21.4%) at high risk of BSI (Figure 3).

**Figure 4:**
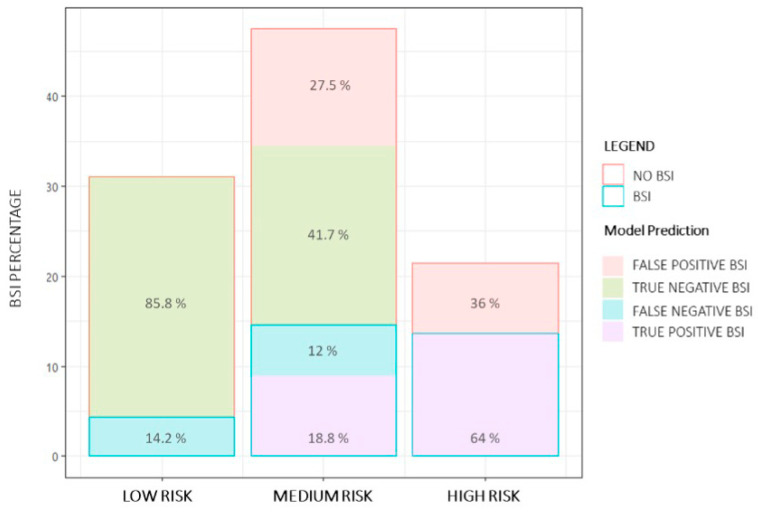
Risk groups with prediction information for positive and negative bloodstream infection (BSI) on the validation set. Positive BSIs are represented by the blue outline, of which the predicted positive ones are represented by the purple fill and the predicted negative ones by the light blue fill. Negative BSIs are represented by the red outline, of which the predicted negative ones are represented by the green fill and the predicted positive ones by the red fill.

The percentages of BSI associated with each risk class were 14.2% (72/508 patients) for low risk, 30.8% (239/776 patients) for medium risk, and 64% (224/350 patients) for high risk. Among low-risk patients in the validation set, 436 patients (85.8%) classified as negative were true negatives, while 72 patients (14.2%) had a BSI and were classified as negatives. In the medium-risk class, 324 patients were true negatives (41.7%), 93 patients were false negatives (12%), 146 patients were true positives (18.8%) and 213 were false positives (27.5%). Among high-risk patients, whom all were predicted as positive, 224 patients (64%) were true positives while 126 patients (36%) had no BSI and were classified as positive (Figure 4).

## 4. Discussion

In the present study, a machine learning-based model was built to predict the probability of having an HA-BSI at the time a BC was requested. On 5660 patients hospitalized from 1 January 2016 to 31 December 2019, for which at least a BC was drawn, the model showed that age > 80 years, fever, hypotension, an altered mental status, the presence of a CVC, BUN > 13 mg/dl, procalcitonin >1 ng/mL, total bilirubin > 2 mg/dl, time from admission to BSI >12 days, 2 or more index comorbidities and platelets < 50,000/mm^3^ were associated with an increased risk of having an HA-BSI. Three risk level groups were identified: low risk, with a BSI prevalence of 14.2%; medium risk, with a BSI prevalence of 30.8%; and high risk, with a BSI prevalence of 64%. The AUROC of the model in the validation set was 0.74, suggesting moderate discriminatory ability. The AUROC result of our study is in line with those reported in other studies, ranging from 0.54 [26] to 0.82 [10]. However, this value, together with the NPV = 0.82, describes a good prediction of true negatives, which is of considerable importance from an antibiotic stewardship point of view. Indeed, the good reliability of true negatives may allow antibiotic therapy not to be administered or, at most, to be delayed in at least 31.1% of patients considered at low risk. Interestingly, the variables included in our model coincide with two of the three major criteria and four of the eight minor criteria of the non-machine learning-based decision rule proposed by Shapiro et al. in 2008 [27], which remains one of the highest-performing predictive models.

As previously noted, physicians tend to overestimate the probability of BSI for many patients [28]. From a practical point of view, assuming to treat all patients for which a BC was required and applying the present model, 86% of antibiotic therapy in patients at low risk would have been saved and delayed in 14% of patients. In the middle-risk group, 41.7% of antibiotic therapies would have been saved but delayed in 12% of patients. In the high-risk group, no antibiotic therapies would have been delayed.

While machine learning-based models built for an early prediction of sepsis are numerous [29,30], especially in Intensive Care Unit (ICU) populations, machine learning-based prediction models of BSI among people for which a BC was required are rare [20]. In a recently published paper, Mahmoud et al. [26] presented a machine learning-based model with a very high specificity but low sensitivity. Unfortunately, almost 90% of the study patients started antibiotic therapy at least 24 h before BC, probably influencing the result. Surprisingly, in the model by Mahmoud et al. [26], procalcitonin level was not correlated to positive BC. In a recent paper, Ratzinger et al. [31] screened 3370 patients with Systemic Inflammatory Response Syndrome (SIRS) in a prospective cohort study and built a random forest model with good performance (AUC 0.738). However, the model did not perform better than procalcitonin alone (AUC 0.729). Other machine learning-based models with high accuracy were built [32], even though only in ICU patients.

Since models such as those presented in this paper are implemented into Electronic Health Records (EHR), real-time processing of the data provides an immediate and seamless calculation of the likelihood of having a BSI. The instant translation of a mathematical model into an explainable and implementable score for clinical decisions enhances its usability in daily practice. This can be especially useful in settings where the healthcare system is overloaded, or decisions need to be made very quickly, such as in the Emergency Department. Machine learning-based models allow us to analyze a large amount of data directly extracted from EHR, overcoming the limitations of many published BSI probability models [33].

A predictive model may assist the clinician in investigating a suspected BSI or might be useful in identifying patients for more expensive diagnostic techniques. However, the present model was not designed as a warning system (detecting the onset of sepsis as early as possible) but as a support for clinicians to decrease unnecessary exposure to antibiotics when the probability of having a BSI is very low. The main driver of antimicrobial resistance (AMR) is the inappropriate use of antimicrobials [7]. According to the antibiotic stewardship perspective, a predictive model of BSI, such as that of the present study, has the potential to support a wise watchful waiting approach [8]. The present model may also contribute to better selecting patients with a high pre-test probability of BSI for whom a BC might be requested [34].

This study has some limitations. First, the model was built on retrospective data from a single clinical center. Even though the amount of analyzed data is relevant, the results of the study cannot be generalized to other clinical centers. Moreover, the study is observational, and the impact of its use on daily clinician decision-making (external validation) was not assessed. Similarly, the cut-offs of the numerical variables that were categorized using thresholds derived directly from the data need external validation. Further studies should evaluate whether the routinary implementation of this model in daily practice may result in significant savings of useless antibiotics and reduction of costs. Finally, due to the variable reliability of data capture within our EHR, we did not include the source of infection, a component correlated to the likelihood of positive BC.

## 5. Conclusions

Our predictive model gives an example of how EHR-based clinical decision support (CDS) systems are promising tools in an antibiotic stewardship approach to thin out unnecessary antibiotic treatments. The study highlights how computational infrastructure and machine learning models, updated in real-time, can continuously inform clinicians of the best clinical decisions, representing a supplement and never replacing the clinical judgment. If the low number of patients with false negative results is confirmed by future studies, clinicians may be supported, in situations of uncertainty or in low-risk patients, not to administer antibiotic therapy or, at most, to delay it. Simple prognostic scores are probably dated, and more advanced multimorbidity models should be considered. We strongly support the 3PM approach: predictive, preventive, and personalized medicine. The availability of tools to prescribe antibiotics more precisely could be a step towards this purpose. Finally, data and models may be shared among centers to refine analyses and improve the fight against antimicrobial resistance using methodology that preserves data ownership and patient privacy [35].

## Figures and Tables

**Table 1:** Characteristics of patients included in the training and validation subsets.

Characteristic	All Patients			Training Subset			Validation Subset		
	N = 5660	BSI (N = 1904)	Non-BSI (N = 3756)	N = 4026	BSI (N = 1369)	Non-BSI (N = 2657)	N = 1634	BSI (N = 535)	Non-BSI (N = 1099)
**Demographics**									
Age, years, median (IQR)	71 (58; 80)	72 (62; 81)	70 (57; 79)	70 (58; 80)	72 (61; 81)	69 (56; 79)	71 (60; 80)	73 (63; 81)	71 (58; 80)
Male (%)	3248 (57.4)	1063 (55.8)	2185 (58.2)	2343 (58.2)	770 (56.2)	1573 (59.2)	905 (55.4)	293 (54.8)	612 (55.7)
Length of stay, days, median (IQR)	19 (12; 30)	25 (16; 41)	17 (11; 26)	20 (13; 32)	27 (17; 43)	18 (11; 27)	17 (11; 27)	22 (14; 35)	15 (10; 23)
Death (%)	1169 (20.7)	562 (29.5)	607 (16.2)	848 (21.1)	410 (29.9)	438 (16.5)	321 (19.6)	152 (28.4)	169 (15.4)
Time to BSI, days, median (IQR)	6 (3; 12)	10 (5; 18)	5 (3; 9)	7 (3; 13)	10 (5; 19)	5 (3; 10)	5 (3; 10)	9 (4; 15.5)	5 (3; 8)
Number of previous hospitalization, median (IQR)	0 (0; 1)	0 (0; 1)	0 (0; 1)	0 (0; 1)	0 (0; 1)	0 (0; 1)	0 (0; 1)	0 (0; 1)	0 (0; 1)
Previous BSI (%)	232 (4.1)	107 (5.6)	125 (3.3)	149 (3.7)	66 (4.8)	83 (3.1)	83 (5.1)	41 (7.7)	42 (3.8)
**Comorbidities**									
Index CM, number, median (IQR)	1 (1; 2)	1 (1; 2)	1 (1; 1)	1 (1; 2)	1 (1; 2)	1 (1; 1)	1 (1; 2)	1 (1; 2)	1 (0; 2)
Diabetes (%)	939 (16.6)	334 (17.5)	605 (16.1)	639 (15.9)	237 (17.3)	402 (15.1)	300 (18.4)	97 (18.1)	203 (18.5)
Immunodepression (%)	945 (16.7)	339 (17.8)	606 (16.1)	646 (16)	226 (16.5)	420 (15.8)	299 (18.3)	113 (21.1)	186 (16.9)
Renal failure (%)	1050 (18.6)	406 (21.3)	644 (17.1)	744 (18.5)	284 (20.7)	460 (17.3)	306 (18.7)	122 (22.8)	184 (16.7)
Neoplasm (%)	2425 (42.8)	859 (45.1)	1566 (41.7)	1699 (42.2)	600 (43.8)	1099 (41.4)	726 (44.4)	259 (48.4)	467 (42.5)
Neurological diseases (%)	993 (17.5)	389 (20.4)	604 (16.1)	756 (18.8)	297 (21.7)	459 (17.3)	237 (14.5)	92 (17.2)	145 (13.2)
**Vital signs**									
Hypoxemia (%)	928 (16.4)	345 (18.1)	583 (15.5)	629 (15.6)	228 (16.7)	401 (15.1)	299 (18.3)	117 (21.9)	182 (16.6)
Dyspnea (%)	581 (10.3)	223 (11.7)	358 (9.5)	418 (10.4)	153 (11.2)	265 (10)	163 (10)	70 (13.1)	93 (8.5)
Fever (%)	4347 (76.8)	1542 (81)	2805 (74.7)	3113 (77.3)	1123 (82)	1990 (74.9)	1234 (75.5)	419 (78.3)	815 (74.2)
Hypotension (%)	1221 (21.6)	541 (28.4)	680 (18.1)	880 (21.9)	392 (28.6)	488 (18.4)	341 (20.9)	149 (27.9)	192 (17.5)
Altered mental status (%)	1857 (32.8)	737 (38.7)	1120 (29.8)	1352 (33.6)	529 (38.6)	823 (31)	505 (30.9)	208 (38.9)	297 (27)
Tachycardia (%)	903 (16)	365 (19.2)	538 (14.3)	624 (15.5)	259 (18.9)	365 (13.7)	279 (17.1)	106 (19.8)	173 (15.7)
**Devices**									
Urinary catheter (%)	3764 (66.5)	1370 (72)	2394 (63.7)	2718 (67.5)	1008 (73.6)	1710 (64.4)	1046 (64)	362 (67.7)	684 (62.2)
Central venous catheter (%)	1707 (30.2)	822 (43.2)	885 (23.6)	1233 (30.6)	594 (43.4)	639 (24)	474 (29)	228 (42.6)	246 (22.4)
**Laboratory**									
White blood cells [WBC], ×10^9^/L ^3^, median (IQR)	9.8 (6.7; 14.1)	9.4 (6.3; 13.7)	10.1 (6.9; 14.3)	10.0 (6.7; 14.3)	9.5 (6.3; 14.0)	10.2 (7.0;14.4)	9.7 (6.6; 13.6)	9.2 (6.4; 13.3)	9.9 (6.8; 13.9)
Neutrophils, ×10^9^/L, median (IQR)	8.0 (5.2; 12.0)	7.9 (5.1; 12.1)	8.1 (5.2; 11.9)	8.3 (5.4; 11.9)	8.3 (5.2; 11.8)	8.4 (5.4; 12.0)	8.0 (5.1; 11.5)	8.0 (5.1; 11.7)	8.0 (5.1; 11.4)
Platelet, ×10^9^/L, median (IQR)	225 (150; 314)	212 (129; 302)	232 (160; 322)	222 (145; 311)	212 (125; 303)	227 (155; 315)	233 (161; 328)	214 (141; 300)	244 (172; 338)
Blood urea nitrogen, mg/dL, median (IQR)	19 (13; 31)	21 (14; 33)	18 (12; 29)	20 (13; 31)	21 (15; 34)	19 (13; 29)	18 (12; 29)	21 (14; 30)	17 (12; 28)
Creatinine, mg/dL, median (IQR)	0.9 (0.7; 1.3)	0.9 (0.6; 1.4)	0.9 (0.7; 1.3)	0.9 (0.7; 1.3)	0.9 (0.6; 1.4)	0.9 (0.7; 1.3)	0.9 (0.7; 1.4)	1.0 (0.6; 1.4)	0.9 (0.7; 1.3)
Total Bilirubin, mg/dL, median (IQR)	0.7 (0.5; 1.3)	0.7 (0.5; 1.5)	0.7 (0.5; 1.2)	0.9 (0.5; 1.7)	1 (0.5; 1.7)	0.9 (0.5; 1.7)	0.8 (0.5; 1.7)	0.9 (0.5; 1.7)	0.8 (0.5; 1.7)
C-reactive protein, mg/L, median (IQR)	123 (59; 187)	116 (59; 184)	126 (58; 188)	135 (66; 176)	132 (67; 171)	135 (64; 178)	131 (60; 186)	123 (55; 188)	135 (63; 186)
Procalcitonin, ng/mL, median (IQR)	0.38 (0.15; 1.62)	0.91 (0.25; 5.22)	0.28 (0.13; 0.89)	0.41 (0.15; 1.71)	0.95 (0.26; 5.33)	0.31 (0.13; 0.94)	0.32 (0.13; 1.41)	0.8 (0.22; 5.19)	0.23 (0.11; 0.75)

BSI, bloodstream infections; IQR, interquartile range; Index CM, comorbidity macro groups; Tachycardia (heart rate > 100/min); Dyspnea (respiratory rate > 20/min); Hypoxemia (SpO2 < 92); Fever (temperature > 37 °C); Hypotension (systolic blood pressure < 90 mmHg).

**Table 2:** Predictors of bloodstream infection (BSI) positivity. Features selected with univariate logistic regression (*p*-value ≤ 0.05 and Odds Ratio > 1) and multivariate logistic regression with stepwise feature selection in five-fold cross-validation.

Variables	Univariate Analysis		Multivariate Analysis		
	*p*-Value	Odds Ratio	Coefficients	95% CI	*p*-Value
Time BSI > 12 days	<0.05	3.42	1.15	0.99–1.31	<0.05
Procalcitonin > 1 ng/mL	<0.05	3.07	1.14	0.99–1.29	<0.05
Presence of a CVC	<0.05	2.42	0.72	0.56–0.88	<0.05
Platelets < 50 × 10^9^/mm^3^	<0.05	2.3	0.59	0.28–0.91	<0.05
Hypotension	<0.05	1.78	0.27	0.09–0.44	<0.05
Blood urea nitrogen> 13 mg/dl	<0.05	1.71	0.35	0.17–0.55	<0.05
Presence of urinary catheter	<0.05	1.55			
Fever	<0.05	1.53	0.72	0.53–0.90	<0.05
Tachycardia	<0.05	1.47			
Altered mental status	<0.05	1.4	0.22	0.05–0.38	0.01
Total bilirubin > 2 mg/dl	<0.05	1.35	0.19	−0.03–0.41	0.09
Index CM ≥ 2	<0.05	1.33	0.25	0.08–0.41	<0.05
Serum creatinine > 3 mg/dl	0.04	1.32			
Age > 80 years	<0.05	1.28	0.36	0.18–0.54	<0.05

BSI, bloodstream infection; CVC, central venous catheter; Index CM, comorbidity macro groups.

## Data Availability

The data that support the findings of this study are available from the corresponding author upon reasonable request.

## Supplementary Materials

The following supporting information can be downloaded at: https://www.mdpi.com/article/10.3390/diagnostics14040442/s1, Table S1: Cut-off numerical variables; Figure S1: Calibration plot on testing data; Figure S2: Lift and gain plot on testing data.

## References

[1] Antimicrobial Resistance Collaborators (2022). Global burden of bacterial antimicrobial resistance in 2019: A systematic analysis. Lancet.

[2] Huerta L.E., Rice T.W. (2019). Pathologic Difference between Sepsis and Bloodstream Infections. J. Appl. Lab. Med..

[3] Singer M., Deutschman C.S., Seymour C.W., Shankar-Hari M., Annane D., Bauer M., Bellomo R., Bernard G.R., Chiche J.D., Coopersmith C.M. (2016). The Third International Consensus Definitions for Sepsis and Septic Shock (Sepsis-3). JAMA.

[4] Maharaj R., McGuire A., Street A. (2021). Association of Annual Intensive Care Unit Sepsis Caseload with Hospital Mortality from Sepsis in the United Kingdom, 2010–2016. JAMA Netw. Open.

[5] Fleischmann-Struzek C., Mellhammar L., Rose N., Cassini A., Rudd K.E., Schlattmann P., Allegranzi B., Reinhart K. (2020). Incidence and mortality of hospital- and ICU-treated sepsis: Results from an updated and expanded systematic review and meta-analysis. Intensive Care Med..

[6] Evans L., Rhodes A., Alhazzani W., Antonelli M., Coopersmith C.M., French C., Machado F.R., Mcintyre L., Ostermann M., Prescott H.C. (2021). Executive Summary: Surviving Sepsis Campaign: International Guidelines for the Management of Sepsis and Septic Shock. Crit. Care Med..

[7] Holmes A.H., Moore L.S., Sundsfjord A., Steinbakk M., Regmi S., Karkey A., Guerin P.J., Piddock L.J. (2016). Understanding the mechanisms and drivers of antimicrobial resistance. Lancet.

[8] Denny K.J., De Waele J., Laupland K.B., Harris P.N.A., Lipman J. (2020). When not to start antibiotics: Avoiding antibiotic overuse in the intensive care unit. Clin. Microbiol. Infect..

[9] Bhavani S.V., Lonjers Z., Carey K.A., Afshar M., Gilbert E.R., Shah N.S., Huang E.S., Churpek M.M. (2020). The Development and Validation of a Machine Learning Model to Predict Bacteremia and Fungemia in Hospitalized Patients Using Electronic Health Record Data. Crit. Care Med..

[10] Zoabi Y., Kehat O., Lahav D., Weiss-Meilik A., Adler A., Shomron N. (2021). Predicting bloodstream infection outcome using machine learning. Sci. Rep..

[11] Tacconelli E., Göpel S., Gladstone B.P., Eisenbeis S., Hölzl F., Buhl M., Górska A., Cattaneo C., Mischnik A., Rieg S. (2022). Development and validation of BLOOMY prediction scores for 14-day and 6-month mortality in hospitalised adults with bloodstream infections: A multicentre, prospective, cohort study. Lancet Infect. Dis..

[12] Jun I., Rich S.N., Marini S., Feng Z., Bian J., Morris J.G., Prosperi M. (2022). Moving from predicting hospital deaths by antibiotic-resistant bloodstream bacteremia toward actionable risk reduction using machine learning on electronic health records. AMIA Jt. Summits Transl. Sci. Proc..

[13] Jin L., Zhao C., Li H., Wang R., Wang Q., Wang H. (2021). Clinical Profile, Prognostic Factors, and Outcome Prediction in Hospitalized Patients with Bloodstream Infection: Results From a 10-Year Prospective Multicenter Study. Front. Med..

[14] Lee C.C., Hung Y.P., Hsieh C.C., Ho C.Y., Hsu C.Y., Li C.T., Ko W.C. (2023). Predictive models for short-term mortality and length of hospital stay among adults with community-onset bacteraemia before and during the COVID-19 pandemic: Application of early data dynamics. BMC Infect. Dis..

[15] Choi D.H., Hong K.J., Park J.H., Shin S.D., Ro Y.S., Song K.J., Kim K.H., Kim S. (2022). Prediction of bacteremia at the emergency department during triage and disposition stages using machine learning models. Am. J. Emerg. Med..

[16] Raita Y., Goto T., Faridi M.K., Brown D.F.M., Camargo C.A., Hasegawa K. (2019). Emergency department triage prediction of clinical outcomes using machine learning models. Crit. Care.

[17] Zhang F., Wang H., Liu L., Su T., Ji B. (2023). Machine learning model for the prediction of gram-positive and gram-negative bacterial bloodstream infection based on routine laboratory parameters. BMC Infect. Dis..

[18] Tsai C.M., Lin C.R., Zhang H., Chiu I.M., Cheng C.Y., Yu H.R., Huang Y.H. (2020). Using Machine Learning to Predict Bacteremia in Febrile Children Presented to the Emergency Department. Diagnostics.

[19] Zhou T., Ren Z., Ma Y., He L., Liu J., Tang J., Zhang H. (2023). Early identification of bloodstream infection in hemodialysis patients by machine learning. Heliyon.

[20] Lee K.H., Dong J.J., Jeong S.J., Chae M.H., Lee B.S., Kim H.J., Ko S.H., Song Y.G. (2019). Early Detection of Bacteraemia Using Ten Clinical Variables with an Artificial Neural Network Approach. J. Clin. Med..

[21] Damiani A., Masciocchi C., Lenkowicz J., Capocchiano N.D., Boldrini L., Tagliaferri L., Cesario A., Sergi P.D., Marchetti A., Luraschi A. (2021). Building an Artificial Intelligence Laboratory Based on Real World Data: The Experience of Gemelli Generator. Front. Comp. Sci..

[22] Stoltzfus J. (2011). Logistic regression: A brief primer. Acad. Emerg. Med..

[23] Mercurio G., Gottardelli B., Lenkowicz J., Patarnello S., Bellavia S., Scala I., Rizzo P., de Belvis A.G., Del Signore A.B., Maviglia R. (2024). A novel risk score predicting 30-day hospital re-admission of patients with acute stroke by machine learning model. Eur. J. Neurol..

[24] Collins G.S., Reitsma J.B., Altman D.G., Moons K.G. (2015). Transparent Reporting of a multivariable prediction model for Individual Prognosis or Diagnosis (TRIPOD). Ann. Intern. Med..

[25] Collins G.S., Dhiman P., Andaur Navarro C.L., Ma J., Hooft L., Reitsma J.B., Logullo P., Beam A.L., Peng L., Van Calster B. (2021). Protocol for development of a reporting guideline (TRIPOD-AI) and risk of bias tool (PROBAST-AI) for diagnostic and prognostic prediction model studies based on artificial intelligence. BMJ Open.

[26] Mahmoud E., Al Dhoayan M., Bosaeed M., Al Johani S., Arabi Y.M. (2021). Developing Machine-Learning Prediction Algorithm for Bacteremia in Admitted Patients. Infect. Drug Resist..

[27] Shapiro N.I., Wolfe R.E., Wright S.B., Moore R., Bates D.W. (2008). Who needs a blood culture? A prospectively derived and validated prediction rule. J. Emerg. Med..

[28] Jeppesen K.N., Dalsgaard M.L., Ovesen S.H., Rønsbo M.T., Kirkegaard H., Jessen M.K. (2022). Bacteremia Prediction with Prognostic Scores and a Causal Probabilistic Network—A Cohort Study of Emergency Department Patients. J. Emerg. Med..

[29] Schinkel M., Paranjape K., Nannan Panday R.S., Skyttberg N., Nanayakkara P.W.B. (2019). Clinical applications of artificial intelligence in sepsis: A narrative review. Comput. Biol. Med..

[30] Fleuren L.M., Klausch T.L.T., Zwager C.L., Schoonmade L.J., Guo T., Roggeveen L.F., Swart E.L., Girbes A.R.J., Thoral P., Ercole A. (2020). Machine learning for the prediction of sepsis: A systematic review and meta-analysis of diagnostic test accuracy. Intensive Care Med..

[31] Ratzinger F., Ratzinger F., Haslacher H., Perkmann T., Pinzan M., Anner P., Makristathis A., Burgmann H., Heinze G., Dorffner G. (2018). Machine learning for fast identification of bacteraemia in SIRS patients treated on standard care wards: A cohort study. Sci. Rep..

[32] Pai K.C., Wang M.S., Chen Y.F., Tseng C.H., Liu P.Y., Chen L.C., Sheu R.K., Wu C.L. (2021). An Artificial Intelligence Approach to Bloodstream Infections Prediction. J. Clin. Med..

[33] Rodic S., Hryciw B.N., Selim S., Wang C.Q., Lepage M.F., Goyal V., Nguyen L.H., Fergusson D.A., van Walraven C. (2023). Concurrent external validation of bloodstream infection probability models. Clin. Microbiol. Infect..

[34] Coburn B., Morris A.M., Tomlinson G., Detsky A.S. (2012). Does this adult patient with suspected bacteremia require blood cultures?. JAMA.

[35] Masciocchi C., Gottardelli B., Savino M., Boldrini L., Martino A., Mazzarella C., Massaccesi M., Valentini V., Damiani A. Federated Cox Proportional Hazards Model with multicentric privacy-preserving LASSO feature selection for survival analysis from the perspective of personalized medicine. Proceedings of the 2022 IEEE 35th International Symposium on Computer-Based Medical Systems (CBMS).

