# Supplementary material for: A Machine Learning Predictive Model of Bloodstream Infection in Hospitalized Patients
Source: Diagnostics (Basel). 2024 Feb 17;14(4):445. doi: 10.3390/diagnostics14040445 (PMC10887662; doi:10.3390/diagnostics14040445)

Table S1: Cut-off numerical variables

**Variables**	**Cut-off**
Age (years)	>80
Blood urea nitrogen, mg/dl	>13
Total Bilirubin, mg/dl,	>1.3; >2; >6
Creatinine, mg/dl,	>3, >0.66
C-reactive protein, mg/l	>184.6
Procalcitonin, ng/ml	>1
White blood cells [WBC], /mm3	>10
Neutrophils, /mm3	>7
Time to BSI (days)	>6; >12
Index_CM	>=2
Platelet, x109/mm3	<150; <100; <50
Cholesterol, mg/dl,	<130

Figure S1: Calibration plot on testing data

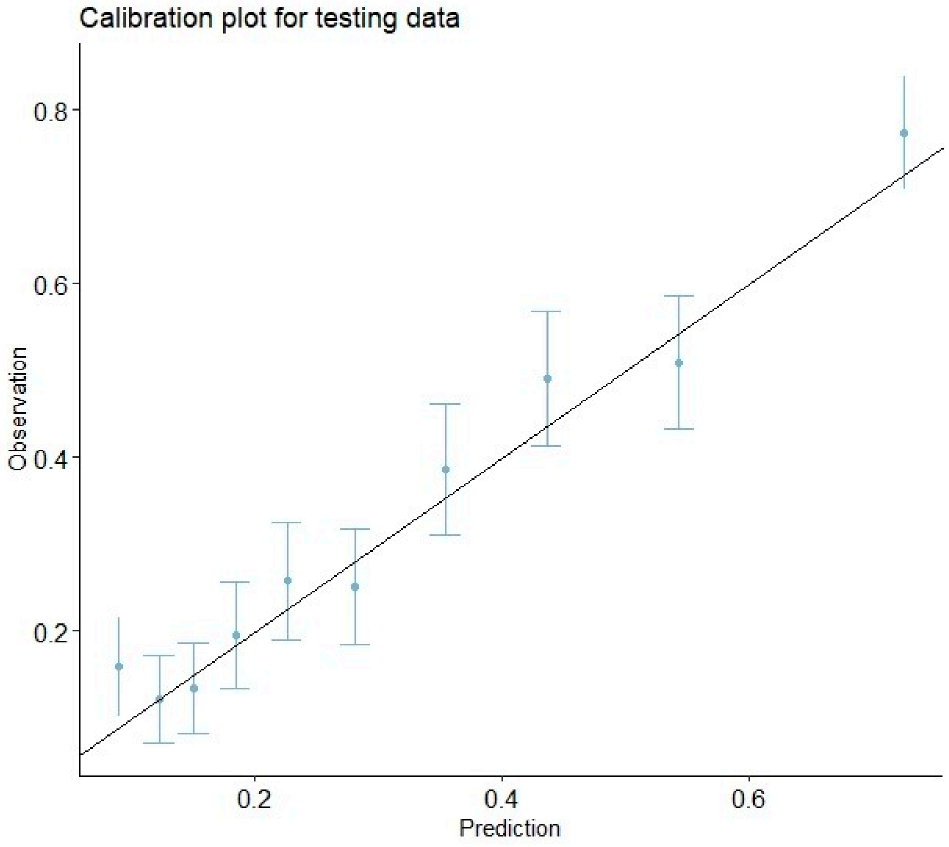

Figure S2: Lift and gain plot on testing data

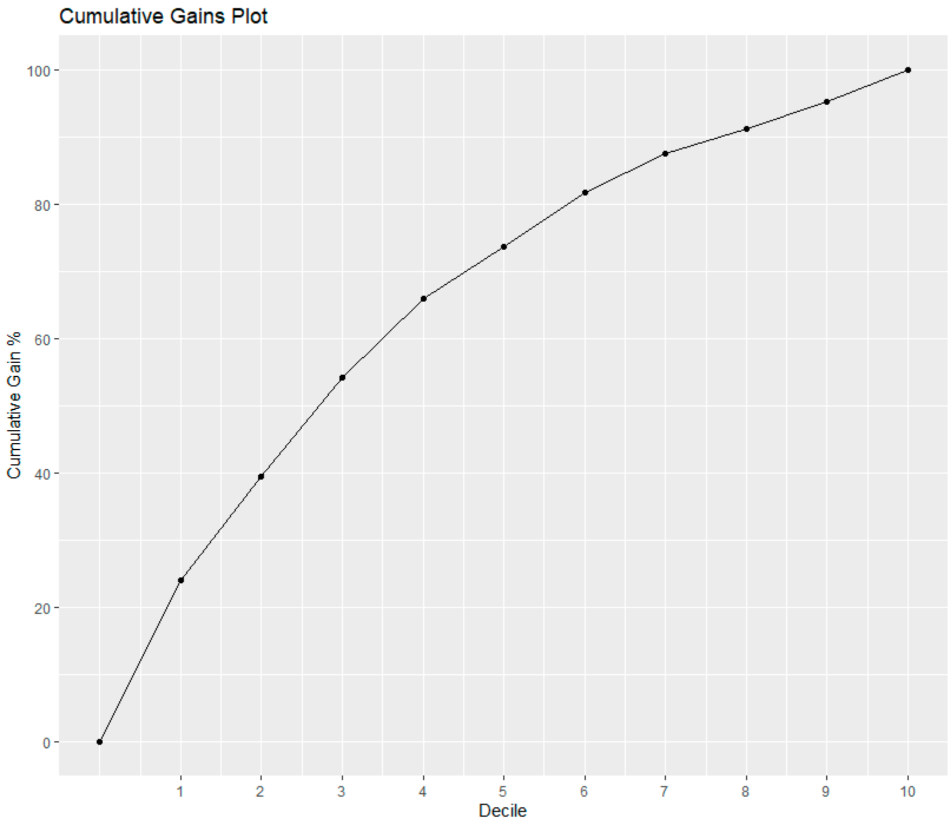

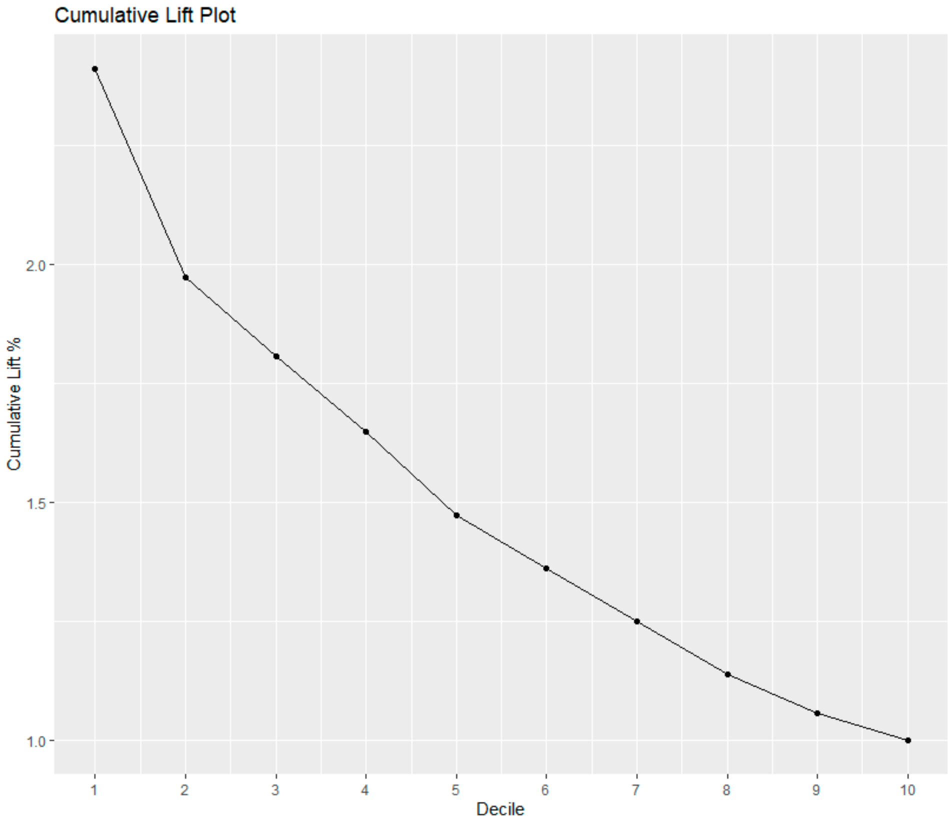

Supplement: Supplementary file 1 [file diagnostics-14-00445-s001.zip › diagnostics-2798691-supplementary.pdf]
